# New water mites of the family Hygrobatidae (Acari, Hydrachnidia) from Turkey

**DOI:** 10.3897/zookeys.361.6389

**Published:** 2013-12-11

**Authors:** Yunus Esen, Vladimir Pešić, Orhan Erman, Yücel Kaya

**Affiliations:** 1 Department of Science Education, Bayburt Faculty of Education, Bayburt University, 69000, Bayburt, Turkey; 2 Department of Biology, Faculty of Sciences, University of Montenegro, 81000 Podgorica, Montenegro; 3 Department of Biology, Faculty of Sciences, Firat University, 23119, Elazığ, Turkey

**Keywords:** Acari, Hygrobatidae, water mites, new records, Turkey

## Abstract

In this study, the findings of three water mite species of the family Hygrobatidae collected from different streams in Turkey were evaluated. *Hygrobates* (s. str.) *anatolicus* Esen & Pešić, **sp. n.** is described as new for science. *Hygrobates (Rivobates) diversiporus* Sokolow, 1927 and *Atractides* (s. str.) *nikooae* Pešić, 2004, which were illustrated and thoroughly discussed, are new records for the Turkish fauna.

## Introduction

After the family Arrenuridae Thor, 1900, the Hygrobatidae Koch, 1842 is the most species-rich in Turkey. So far, 42 species have been found in Turkey (Erman et al. 2010, Esen et al. 2013) belonging to the genera *Atractides* Koch, 1837 (31 species), *Hygrobates* Koch, 1837 (9 species) and *Mixobates* Thor, 1905 (2 species).

During a survey of the freshwater fauna of Kahramanmaraş, Malatya and Siirt Provinces, Turkey, three interesting species for the Turkish fauna were collected. This article aims to describe this material and contribute to our knowledge of water mites distribution in Turkey.

## Material and methods

During fieldwork, water mites were collected by hand netting, sorted on the spot from the living material, conserved in Koenike’s fluid and dissected as described elsewhere (e.g., Gerecke et al. 2007). The holotype and some paratypes of the new species are deposited in the research collection of the Department of Biology, Fırat University, Elazığ, Turkey, other paratypes are deposited in the Museum of Natural History of Montenegro, Podgorica, Montenegro.

The composition of the material is given as: (males/females/deutonymphs). All measurements are given in micrometers. For a detailed description and discussion of the characteristics of the genus *Atractides* and a detailed methodological introduction, see Gerecke (2003) and Davids et al. (2005). The following abbreviations are used: asl. = above sea level, Ac-1 = first acetabulum, Cx-I = first coxae, dL = dorsal length, H = height, L = length, %L = relative length, I-L-6 = Leg 1, sixth segment (tarsus), mL = medial length, P-1 = palp, first segment, S-1= large proximal ventral seta at I-L-5, S-2 = large distal ventral seta at I-L-5, Vgl = ventroglandulare, V = ventrale, W = width.

## Results

### Family Hygrobatidae Koch, 1842

#### Genus *Hygrobates* Koch, 1837

##### Hygrobates (s. str.) anatolicus

Esen & Pešić sp. n.

http://zoobank.org/AE8FEF58-1CEE-4F5F-BFC7-EFEFBFC71BE6

http://species-id.net/wiki/Hygrobates_anatolicus

Figs 1 , 2A–C, F, I

###### Material examined.

Holotype: male, dissected and slide mounted in Hoyer’s fluid, Turkey: Kahramanmaraş Province, Çağlayancerit, Göksu stream, 37°44'26"N, 37°22'21"E, 975 m asl., 28.10.2010. Paratypes: 33/49/0, same data as holotype, five males and five females dissected and slide mounted in Hoyer’s fluid.

###### Diagnosis.

Integument lineated. P-2 ventral margin straight, distally forming a right angle; P-4 ventral setae at the same level.

###### Description.

General features: Integument lineated, occassionaly lines formed as irregular ridges (Fig. 2C). Posteromedial margin of Cx-I slightly triangular, Cx-IV medial margin nose-like protruding. Acetabula arranged in an obtuse triangle; excretory pore unsclerotized, distance genital field – excretory pore L in male 110-241, in female 280-351. Palp: P-2 ventral margin straight, distally forming a right angle, denticles covering two-thirds of the ventral margin of both P-2 and P-3; P-4 ventral setae on the same level.

**Figure 1.**
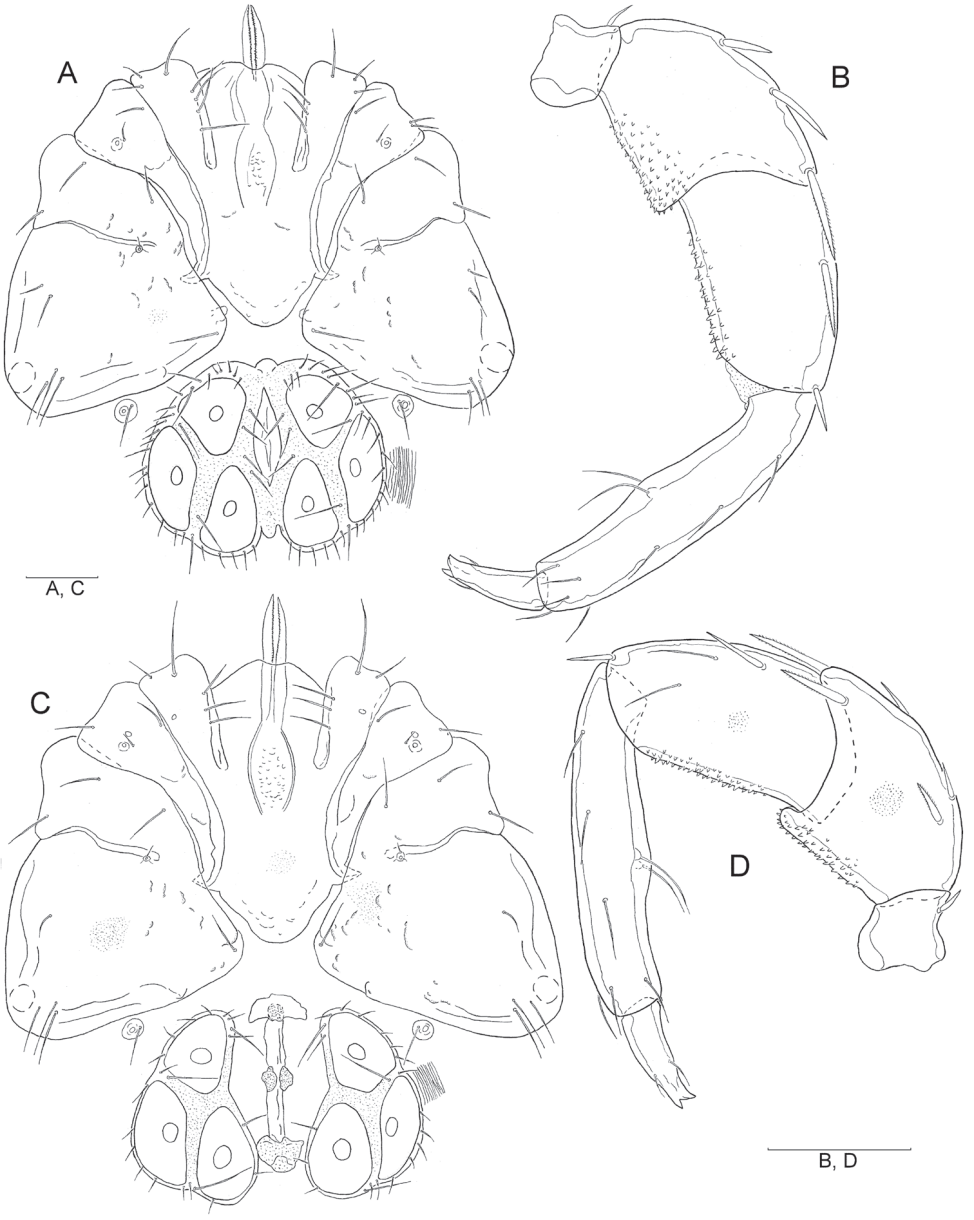
**A–D** *Hygrobates (s. str.) anatolicus* sp. n. (**A–B** male **C–D** female): **A** Coxal and genital field **B** Palp, medial view **C** Coxal and genital field **D** Palp, lateral view (Scale bars = 100 µm).

**Figure 2.**
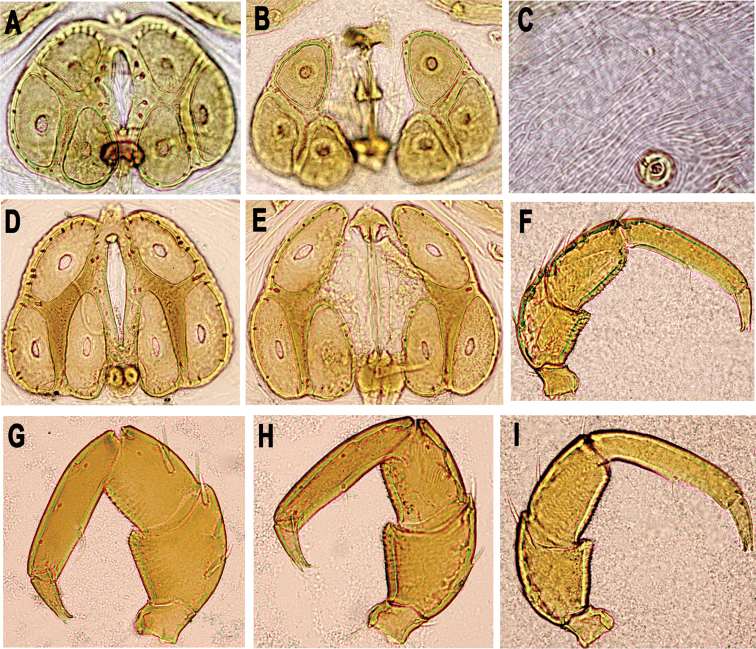
**A–C, F, I** photographs of *Hygrobates anatolicus* sp. n. (**A, F** male **B–C, I** female), Göksu stream, Turkey **D–E, G–H** photographs of *Hygrobates nigromaculatus* Lebert, 1879 (**D, G** male **E, H** female), Ohrid Lake, Macedonia: **A–B, D–E** genital field **C** detail of dorsal integument **F–I** palp.

Male (holotype, in parentheses measurements of paratype, n = 5): Idiosoma L/W 960/810 (720–1115/645–940); coxal field (Fig. 1A) L/W 516/680 (495–612/600–745), median length of Cx-I + gnathosoma 395 (380–450); genital plate (Figs 1A, 2A) L/W 261/340 (210–285/315–380), gonopore L 137 (108–130), L Ac-1–3: 107 (102–112), 145 (140–150), 121 (115–125); anterior margin with a small, knob-shaped medial projection, posterior margin indented, with a short, rounded medial projection. Distance between genital field and excretory pore L 200 (110–241). Palp (Fig. 1B, 2F) total L 621 (586–665), dL: P-1, 40 (36–48); P-2, 157 (146–170); P-3, 136 (128–140); P-4, 218 (208–235); P-5, 70 (68–72). Chelicera L 487 (440–496), claw L 170 (157–172). Legs: dL of I-L-4–6: 257 (250–270), 266 (258–275), 243 (235–258); dL of IV-L-4–6: 391 (365–410), 397 (382–422), 346 (325–368).

Female (n = 5): Idiosoma L/W 720–1507/540–1250; coxal field (Fig. 1C) L/W 495–610/550–847; median length of Cx-I + gnathosoma 400–460. Palp (Figs 1D, 2I) total L 668–749, dL: P-1, 47–51; P-2, 160–198; P-3, 144–160; P-4, 241–262; P-5, 78–80. Chelicera L 490–548, claw L 170–190. Genital field (Fig. 1C, 2B) W 330–418, genital plate L 230–268, genital opening L 210–280, L Ac-1–3: 110–120, 145–150, 126–130. Legs: dL of I-L-4–6 285–302, 295–310, 267–286; dL of IV-L-4–6: 430–456, 440–460, 361–385.

###### Discussion.

Due to the shape of palp with a straight ventral margin of P-2, distally forming a right angle, the new species closely resembles *Hygrobates (s. str.) nigromaculatus* Lebert, 1879 (Fig. 2D–E, G–H) and *Hygrobates setosus* Besseling, 1942. The later species, for a long time was considered a morphotype of *Hygrobates nigromaculatus* (Viets 1960), but differs in size (median length of Cx-I + gnathosoma > 350 µm. Males: P-4 length > 140, genital plate length > 170 µm. Females: P-4 length > 165, genital plate length > 175 µm), life cycle with larvae parasitic on chironomid Diptera and habitat preference for running waters (Martin et al. 2010). The larger dimensions and habitat preference for running waters makes the new species close to *Hygrobates setosus*. However, presence of lineated integument will easily distinguished *Hygrobates anatolicus* sp. n. from two above-mentioned species bearing finely striated integument.

###### Remarks.

Due to the shape of the genital field, population from Göksu stream resembles populations of *Hygrobates nigromaculatus* and *Hygrobates setosus* from the Northern Germany (P. Martin pers. communication). However, population of *Hygrobates nigromaculatus* from the Ohrid Lake clearly differs in the shape of genital field (see Figs 2D-E), with the acetabula distinctly elongated, similar to those in *Hygrobates longiporus* Thor, 1898. The similar, *longiporus*-shape of the acetabula was recently detected in the population of *Hygrobates nigromaculatus* from Luxembourg (R. Gerecke pers. communication), suggesting that this character, in the *Hygrobates nigromaculatus* like-species complex, vary and can not be used in taxonomical separation. If possible the species should be included in a possibly molecular and morphological revision of the *Hygrobates nigromaculatus* like-species complex.

###### Etymology.

Named after the country of the type locality.

###### Habitat.

Rhithrobiont.

###### Distribution.

Known only from the type locality in Kahramanmaraş Province, Turkey.

##### Subgenus *Rivobates* Thor, 1897

###### Hygrobates (Rivobates) diversiporus

Sokolow, 1927

http://species-id.net/wiki/Hygrobates_diversiporus

Figs 3 , 4

####### Material examined.

Turkey,Malatya Province, Doğanşehir, Avcapınar stream, 38°00'38"N, 37°57'56"E, 1335 m asl., 04.07.2004, (7/24/0).

####### Compared material.

Senckenberg Museum Frankfurt, Germany, *Hygrobates (Decabates) quanaticola*, holotype, ♂, P.J/15, Locality. Quanat near Rezazeh, 29.9.1974 coll. Schwoerbel; präp. J/14, *Hygrobates (Decabates) quanaticola*, ♀, Quanat near Rezazeh, 29.9.1974, Schwoerbel.

####### Morphology.

General characters. Posteromedial margin of Cx-I convexly rounded, medial margin of Cx-IV rounded; genital field with 8–13 pairs of acetabula. Ventral margin P-2 proximally concave, distally protruding in a nose- or knob-shaped projection bearing denticles, distal part of P-3 ventral margin covered by denticles, P-4 ventral setae distance 14–19 µm.

**Figure 3.**
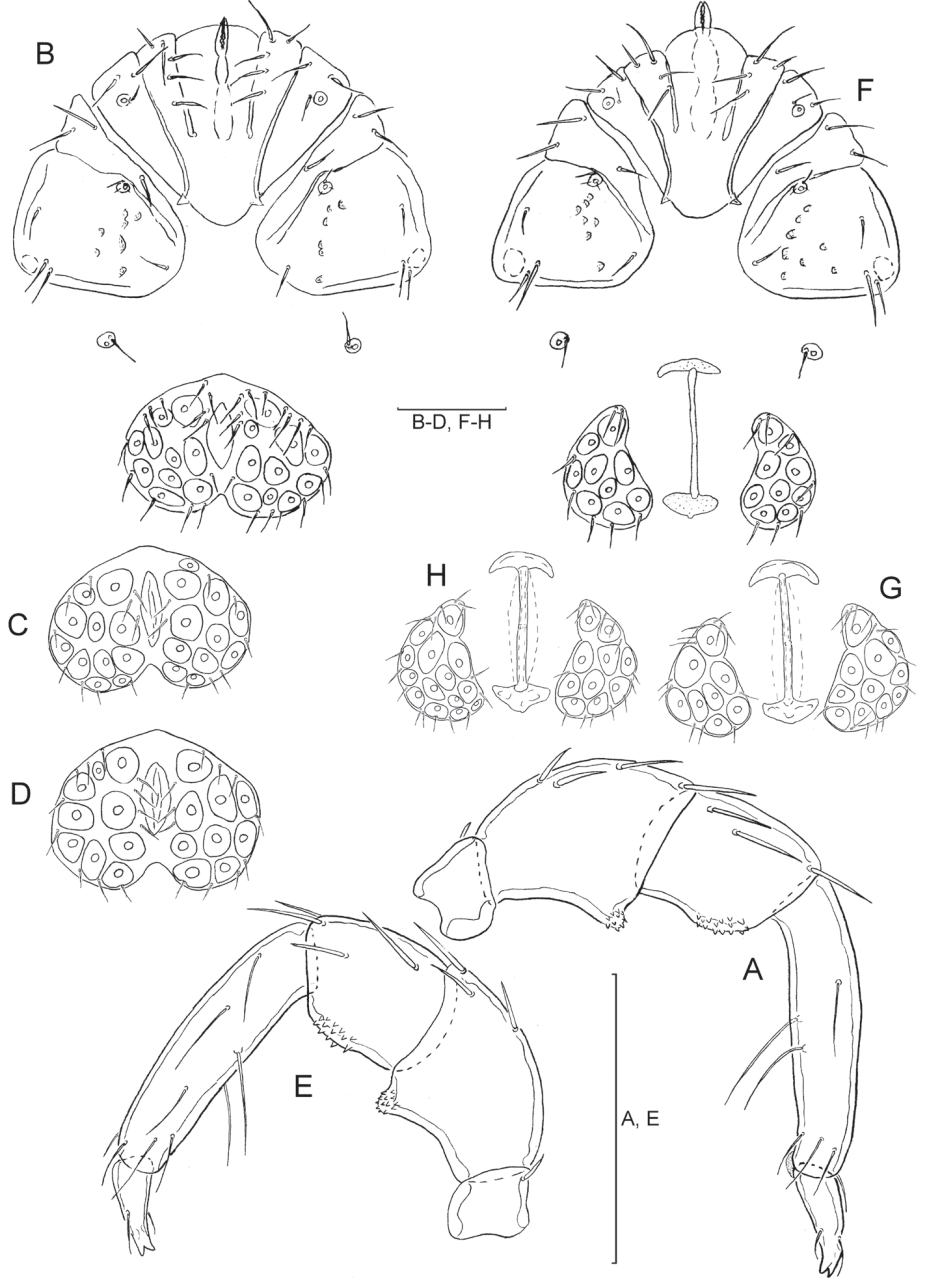
**A–H** *Hygrobates (Rivobates) diversiporus* Sokolow, 1927 (**A–D** male **E–H** female): **A** Palp, medial view **B, F** Coxal and genital field **C–D, G–H** Genital field **E** Palp, lateral view (Scale bars = 100 µm).

Male (n =3):Idiosoma L 805–890 W 690–783; median length of Cx-I + gnathosoma 232–240. Genital field (Figs 3B–D) L 188–191, W 242–273, posterior margin strongly indented. Gonopore L 88–90, distance between genital field and excretory pore 72–100. Palp (Fig. 3A): total L 366–388, dL: P-1, 30–32; P-2, 96–104; P-3, 69–70; P-4, 130–140; P-5, 41–42; chelicera L 210–225.

Female (n =5): Idiosoma L 815–1058 W 670–910; median length of Cx-I + gnathosoma 243–248; genital plate (Figs 3F–H) L 167–180, W 100–104. Distance between genital field and excretory pore L 83–110, genital opening L 200–250, maximum diameter of egg 170. Palp (Fig. 3E): total L 374, dL: P-1, 32–35; P-2, 100–103; P-3, 70–72, P-4, 140–142; P-5 43–45; chelicera L 225–247.

####### Remarks.

Sokolow (1927) described *Hygrobates diversiporus* based on one male and one female specimen from a first order stream in Caucasus. Later on this species has been reported by Bader (1955) from the Ohrid Lake in Macedonia. The latter record of this probably rithrobiontic species from a lacustrine habitat, require confirmation for a better understanding of its geographical distribution. The specimens from Turkey agree well with the type specimen in the shape of male genital field orginally desribed by Sokolow (1927) in German as reverse heart-shaped (“verkehrt-herzförmig”), with an acute anterior angle and a indented posterior margin having a broad, rounded median notch.

**Figure 4.**
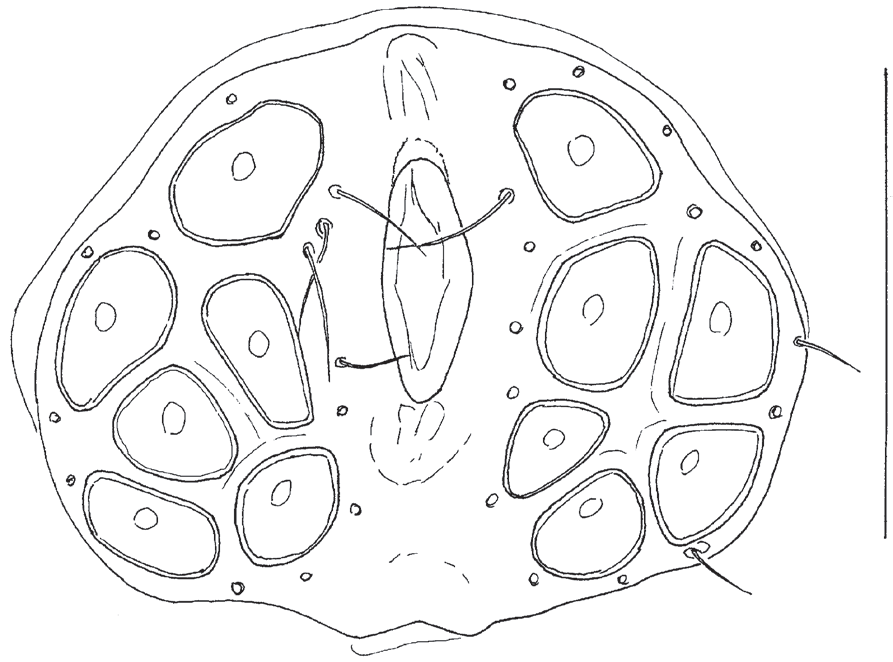
*Hygrobates quanaticola* Schwoerbel & Sepasgozarian, 1976, male holotype: genital field (Scale bar = 100 µm).

The second member of subgenus *Rivobates* Thor known from Turkey, *Hygrobates quanaticola* Schwoerbel & Sepasgozarian, 1976, has been orginally described from Iran (Schwoerbel and Sepasgozarian 1976), and later on reported from Kayseri, Elazığ and Afyon provinces in Turkey (Erman et al. 2010). This species differs (based on re-examination of the holotype) from *Hygrobates diversiporus* in the shape of male genital field with irregularly convex posterior margin (compare Figures 3B–D and 4).

####### Habitat.

Rhithrobiont.

####### Distribution.

Russia (Caucasus). New for Turkey.

#### Genus *Atractides* Koch, 1837

##### Atractides (s. str.) nikooae

Pešić, 2004

http://species-id.net/wiki/Atractides_nikooae

Figs 5 , 6

###### Material examined.

Siirt Province, Kezer stream, 37°57'42"N, 41°51'25"E, 545 m asl., 16.09.2012, (4/8/0); Başur stream, 37°57'42"N, 41°47'19"E, 525 m asl., 15.09.2012, (0/2/0).

###### Morphology.

General features. Integument dorsally finely striated; muscle attachment plates unsclerotized. Coxal field: mediocaudal margin of Cx-I+II with a slightly concave or convex area between the laterally directed apodemes of Cx-II. Palp: weak sexual dimorphism, P-2 and P-3 ventral margin straight; P-4 with maximum height near proximoventral hair, sword seta near distoventral hair, ventral margin divided by hair insertions 1:1:1. Genital field with Ac in a weakly curved line; excretory pore smooth; Vgl-1 separate from Vgl-2. I-L-5: S-1 and -2 strongly heteromorphic and widely distanced, S-2 strongly thickened in the basal third; I-L-6 strongly curved, basally thickened.

**Figure 5.**
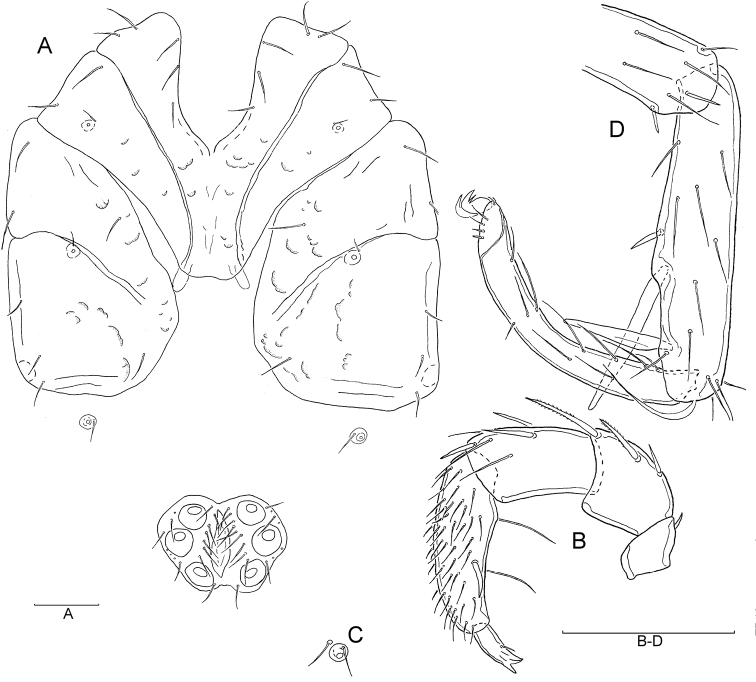
**A–D** *Atractides (s. str.) nikooae* Pešić, 2004, male: **A** Coxal and genital field **B** Palp, medial veiw **C** Vgl-1–2 **D** I–L-5–6 (Scale bars = 100 µm).

Male (n = 2). Idiosoma L 470–527 W 420–432. Coxal field (Fig. 5A) L 320–311, Cx-III W 340–360, Cx-I+II medial suture line L 105–108. Palp (Fig. 5B) total L 285–296, dL and %L (in parentheses): P-1, 26–28 (9.0–9.5); P-2, 64–67 (22.2–22.6); P-3, 68–70 (23.6–23.7); P-4, 97–100 (33.8); P-5, 30–31 (10.5); chelicera L 170–187. Genital field apple shaped, L 90–92, W 100, anterior and posterior margin with shallow indentations (Fig. 5A). Legs: I-L-5 dL 192–193, vL 110–113, H 45–47; S-1 L 95, S-2 L 66–68; S-1–2 interspace 40–42; I-L-6 L 160–165, H 22–23; dL ratio I-L-5/6 1.2.

**Figure 6.**
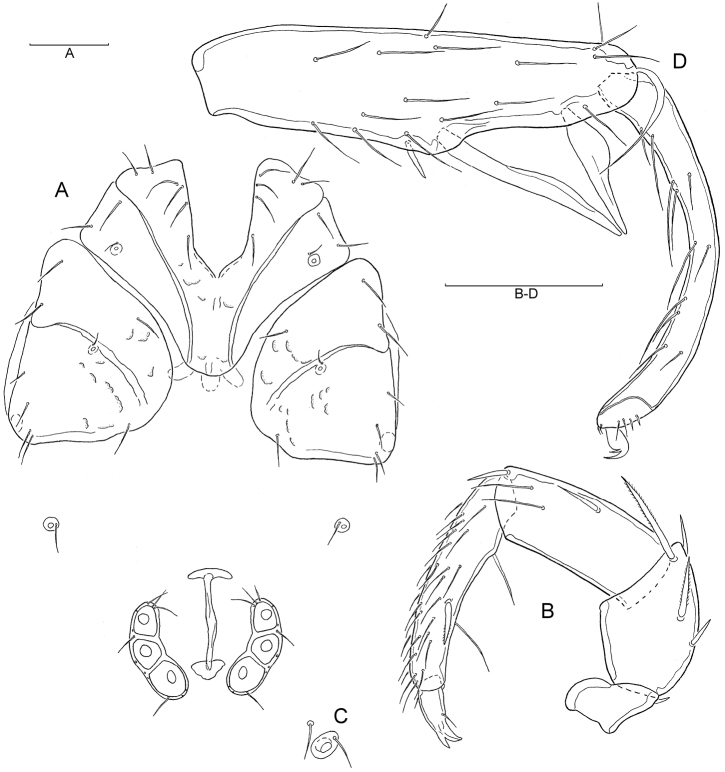
**A–D** *Atractides (s. str.) nikooae* Pešić, 2004, female: **A** Coxal and genital field **B** Palp, medial veiw **C** Vgl-1–2 **D** I–L-5–6 (Scale bars = 100 µm).

Female (n = 5). Idiosoma L 745–760 W 640–652. Coxal field (Fig. 6A) L 382–421, Cx-III W 465–480, Cx-I+II mL 135–142. Palp (Fig. 6B) total 417–445, dL and %L (in parentheses): P-1, 38–40 (9.0); P-2, 90–100 (22.1); P-3, 107–116 (30.0); P-4, 142–148 (33.4); P-5, 40–41 (9.5); P-4 more slender than in male; chelicera L 208. Genital field W 180–204, genital plate L 110–121. Legs: I-L-5 dL 263–280, vL 140–148, H 70–76; S-1 L 128–138, S-2 L 72–88, W 20–21 (ratio 3.6–4.2), ratio L S-1/2 1.78–1.57, S-1–2 interspace 70–72; I-L-6 L 217–230, H 28–30; dL ratio I-L-5/6 1.2.

###### Remarks.

Due to the similar morphology of the genital field (relatively small Ac arranged in a weakly curved line, male genital field apple-shaped with anterior an posterior margin slightly indented), I-L-5 and -6 (S-1 and S-2 with relatively wide setal interspace, I-L-6 strongly curved and slender) and palp (without sexual dimorphism, P-2 ventral margin straight in the both sexes), the specimens from Turkey shows conformity with *Atractides nikooae* Pešić, 2004, a species known from both sexes from the Markazi Province (western Iran, Pešić et al. 2004).

*Atractides (s. str.) diastema* (Szalay, 1935), a weakly defined species from Hungary and Poland, known only from a female sex, differs from *Atractides nikooae* (in parentheses data taken from Gerecke 2003) in a weakly S-shaped ventral margin of P-2, ventral margin P-4 divided by hair insertions in sections 2:2:1, more stouter palp segments (L/H P-3 2.77, P-4 4.2 ), and a less heteromorphic setae S-1/2 (L S-1/2 1.3).

###### Habitat.

Rhithrobiont.

###### Distribution.

Iran (Pešić et al. 2004). New for Turkey.

## Supplementary Material

XML Treatment for Hygrobates (s. str.) anatolicus

XML Treatment for Hygrobates (Rivobates) diversiporus

XML Treatment for Atractides (s. str.) nikooae
